# Nitrendipine and Dementia: Forgotten Positive Facts?

**DOI:** 10.3389/fnagi.2018.00418

**Published:** 2018-12-18

**Authors:** Michal Novotny, Blanka Klimova, Martin Valis

**Affiliations:** ^1^Biomedical Research Centrum, University Hospital Hradec Králové, Hradec Králové, Czechia; ^2^Department of Neurology, University Hospital Hradec Králové, Hradec Králové, Czechia; ^3^Faculty of Informatics and Management, University of Hradec Králové, Hradec Králové, Czechia

**Keywords:** dementia, Alzheimer disease, prevention, calcium channel blockers, nitrendipine

## Abstract

Nowadays, there are about 50 million people suffering from dementia worldwide. In 2030, it is expected that there will be 82 million people living with dementia and in 2050, their number should reach 152 million. This increase in the number of people with dementia results in significant social and economic problems. Therefore, researchers attempt to reduce risk factors causing the development of dementia such as high blood pressure. Epidemiological studies have shown that hypertension increases the risk of dementia at an older age. It can, therefore, be assumed that hypertension therapy will reduce the risk of dementia. However, previous clinical studies have shown that the efficacy of different antihypertensive drugs differs in this respect. The drug group that appears to be the most effective in these analyses is calcium channel blockers (CCBs). The most significant preventive efficacy in terms of protection against dementia has been demonstrated with nitrendipine. Its use is, therefore, particularly advantageous in elderly patients with systolic hypertension who are at high risk of dementia. The purpose of this study is to restore the discussion on the prevention of vascular dementia and Alzheimer’s dementia with nitrendipine in indicated hypertonic patients. The authors performed a literature search of available sources describing the issue of dementia, hypertension and its treatment with nitrendipine. In addition, they made a comparison and evaluation of relevant findings. The results of the detected research studies indicate that nitrendipine is able to reduce the incidence of dementia [Alzheimer’s disease (AD), vascular and mixed] by 55%. The treatment of 1,000 patients with nitrendipine for 5 years may prevent 20 cases of dementia. However, what has not yet been explained is the temporal link between hypertension and dementia due to the long-time intervals between hypertension and the development of dementia.

## Introduction

Currently, there is a growing number of the elderly worldwide. It is estimated that by 2050, the number of older people will reach 1.5 billion, which is twice as much as at present (see Klimova and Kuca, 2016). This trend in the increase of older people is then associated with a rise of age-related diseases such as dementia. Nowadays, there are about 50 million people suffering from dementia worldwide. In 2030, it is expected that there will be 82 million people living with dementia and in 2050, their number should reach 152 million (WHO, 2017). This increase in people with dementia results in significant social and economic problems (Klimova et al., 2015; Maresova et al., 2016). For instance, the total global societal cost of dementia in 2015 was US$ 818 billion, which equals to 1.1% of global gross domestic product (WHO, 2017).

Dementia is generally defined as a sustained deterioration of mental abilities, which affects performance of daily activities (Morris, 1996; Klimova et al., 2016). It is a brain disorder, usually caused by changes in the brain tissue (Nieoullon, 2011). Clinically, dementia is mainly connected with deterioration of function in the association cortex. Pharmacologically and pathologically, abnormalities are more dispersed and expand into sensorimotor cortical areas (Plum, 1986). The main symptoms of dementia include memory loss, orientation problems, impaired communication skills, depression, behavioral changes, or confusion (Klimova and Kuca, 2016). The most common type of dementia is Alzheimer’s disease (AD), which accounts for 60%–80% of all dementia cases. The second most frequents type is Vascular dementia (17% of all dementia cases), and the third one is dementia with Lewy bodies (10%–25% of all dementia cases). Other types of dementia involve Parkinson’s disease dementia (PDD), frontotemporal dementia/degeneration (FTD), and mixed dementia (Alzheimer’s Association, 2015).

The causes of dementia are different, but the main ones include: stroke and ischemic encephalopathy (multi-infarct or vascular dementia), hippocampal sclerosis, head trauma, hydrocephalus, CNS infections, metabolic CNS disorders, demyelinating diseases (multiple sclerosis), neurodegenerative diseases (AD or PDD), neuropsychiatric disorders, severe medical illness or organ failure, or the effects of medications (Agamanolis, 2012).

There are several risk factors (age, family history, or diabetes), which contribute to the development of dementia. High blood pressure is another of the major risk factors for developing dementia at an older age (Durgan and Bryan, 2012; Elias et al., 2012; Faraco and Iadecola, 2013). The relationship between the treatment of hypertension and reduction of the risk of impairment of cognitive function and dementia is sought. As clinical trials have shown, treating hypertension reduces the risk of dementia, but the efficacy of various antihypertensives in their effect on dementia varies (Skinner et al., 1992; Maxwell et al., 1999; Bachmeier et al., 2011; Paris et al., 2011). The first study, which demonstrated a reduction in the incidence of dementia in the treatment of hypertension, was the SYST-EUR study. This was a prospective, double-blind study comparing the treatment of isolated systolic hypertension with calcium channel blocker (CCB) nitrendipine to placebo. Subsequent repeated results have been further refined, and nitrendipine has taken a unique place among CCBs (Staessen et al., 1997; Forette et al., 1998, 2002; Bachmeier et al., 2011; Paris et al., 2011). Thus, the most significant preventive efficacy in terms of protection against dementia seems to be provided by nitrendipine. Its use is particularly advantageous in elderly patients with systolic hypertension who are at high risk of dementia. The authors aim to restore the discussion on the prevention of vascular dementia and Alzheimer’s dementia with nitrendipine in indicated hypertonic patients.

In the near future with increasing life expectancy and occurrence of mainly systolic hypertension, dementia will be not only a serious health problem. The treatment of hypertension may affect dementia in both primary and secondary prevention.

## Methods

The authors performed a literature review of available studies on the research topic describing different types of dementia, its symptoms, causes, and treatment, as well as the effect of nitrendipine on the treatment of hypertension in people aged 60 or more. Research studies were selected on the basis of research topics such as dementia, hypertension, high blood pressure, nitrendipine, and treatment found in the world’s acknowledged databases Web of Science, PubMed, Springer and Scopus. The search was not limited by any period. From the database/journal searches, 5,137 titles/abstracts were retrieved. The majority of the studies were detected in the PubMed database. The titles and abstracts of some identified articles were then checked for relevance. Subsequently, the search was performed again, focusing on the occurrence of at least one keyword in the title or abstract, thereby significantly narrowing the selection. It provided the authors with a relevant entry-level file base. Altogether, 326 studies were found. After removing duplicates and titles/abstracts unrelated to the research topic, 33 English-written studies were found relevant to the research topic. The information found in the selected studies on dementia, its epidemiology, pathophysiology, symptoms, diagnosis, hypertension, nitrendipine, and treatment was carefully evaluated and it is described and discussed in the following sections.

## Association Between Hypertension and Dementias

There is a consensus in the literature on the direct influence of hypertension on cognitive impairment; hypertension is associated with impaired memory, lack of attention, slow processing of information and perceptions, and reduced abstract thinking (Elias et al., 2012). One of the first studies dealing with the relationship between the high blood pressure and cognitive decline was the Framingham study (Farmer et al., 1990; Elias et al., 1993). Subsequently, other studies were conducted, for example, the Rotterdam study (Ruitenberg et al., 2001), the Kungsholmen study (Guo et al., 1996), Honolulu—Asia Aging Study (Launer et al., 2000), Epidemiology of Vascular Aging Study (Morris et al., 2000). Studies have consistently come to the conclusion that arterial hypertension is associated with a slight decline in cognition. In summary, clinical trials show that a decrease in blood pressure of both systolic and diastolic by 10 mm Hg significantly reduces the risk of conversion of mild cognitive defect to dementia (Ravaglia et al., 2006; Dickstein et al., 2010; Hanon, 2010; Sierra et al., 2012). The exact mechanism of cognitive decline in relation to arterial hypertension is not entirely clear. One hypothesis states that microcirculatory vascular damage due to increased blood pressure is being involved in the mechanism of cognitive decline (Ravaglia et al., 2006; Dickstein et al., 2010). Another significant risk factor affecting circulatory vascular damage is chronic intermittent hypoxia as a consequence of sleep apnea. This hypoxia increases blood pressure, induces brain endothelial dysfunction, and suppresses the increase in blood flow to the brain. This, in turn, results in a lack of energy substrates for active brain tissue (Capone et al., 2012; Durgan and Bryan, 2012). Another possible mechanism is linked to calcium channels and the entry of calcium into nerve cells. Calcium accumulation in neurons increases with age, and can be the trigger mechanism of degenerative processes of nerve tissue. In addition, for Alzheimer’s dementia, there is evidence that hypertension can promote the accumulation and aggregation of beta amyloid in the brain (Cummings, 2004; Rodrigue et al., 2013). Similarly, experimental data show that vascular clearance of beta amyloid is impaired in dysfunctional cerebral blood vessels. All this is likely to lead to amyloid angiopathy and subsequent dysfunction of the blood-brain barrier (Faraco and Iadecola, 2013; Park et al., 2013).

However, what has not yet been explained is the temporal link between hypertension and dementia. On the one hand, there are studies demonstrating lower blood pressure in people with dementia (Tzourio et al., 2014); on the other hand, there are long-term observational studies where dementia patients have a history of increased blood pressure (Skoog et al., 1996). In randomized clinical trials, it is still a great challenge for scientists to demonstrate that hypertension treatment reduces the risk of developing dementia at a later age. The main complications in this case are long-time intervals between the hypertension and the onset of dementia (Staessen et al., 2011).

## Calcium Channel Blockers (CCBs)

CCBs can be divided into three generations: (a) I. generation (nifedipine, verapamil, diltiazem); (b) II. generation (isradipine, felodipine, nitrendipine, nisoldipine); and (c) III. generation (amlodipine). From the point of view of the chemical structure, CCB can be divided into non-dihydropyridines and dihydropyridines (Elliott and Ram, 2011).

Based on available clinical data, CCBs have been shown to have their place in a number of indications (Tijssen and Lubsen, 1988; Deanfield et al., 1994; Held and Yusuf, 1994; Gibson and Boden, 1996; Rehnqvist et al., 1996; Pitt et al., 2000). In patients with ischemic heart disease, their antianginal effect is dominant. According to some studies (e.g., PREVENT, Pitt et al., 2000), apart from symptomatic relief, there has been a beneficial effect on morbidity. Available data suggest that reducing the risk of stroke is better with CCB therapy than with other first-line treatment groups, but with a slightly lower risk of myocardial infarction. Combined indicators of morbidity and mortality are, however, affected in a comparable way (Tijssen and Lubsen, 1988; Held and Yusuf, 1994; Gibson and Boden, 1996). The dihydropyridine CCBs bind in those places that are affected by AD the most (Hockerman et al., 1997; Nimmrich and Eckert, 2013).

How would CCB, in addition to their antihypertensive effect, affect cognitive function, or AD? Free intracellular calcium is one of the most important messengers for many signal transduction pathways of neurons. Any disturbance in the level of this ion seems to result in the changes of the brain aging processes, memory deficits, and cell death. Calcium homeostasis involves four types of voltage-controlled Ca2^+^ channels (T, L, P/Q, N). Type L is primarily found on neurons and is affected by CCBs. Therefore, the blockade of these L-channels, along with direct interaction with NMDA receptors, could be one of the main mechanisms of the effects of nitrendipine (Sen et al., 1993; Hockerman et al., 1997). Based on these facts, the “calcium hypothesis” of AD was set—Ca^2+^ homeostasis disorders are the most likely cause of neurodegenerative changes in AD (LaFerla, 2002; Yasar et al., 2005). In AD, enhanced calcium load may be caused by extracellular accumulation of amyloid-β (LaFerla, 2002; Yasar et al., 2005). Research studies indicate that soluble forms accelerate the influx through calcium-conducting ion channels in the plasma membrane, leading to excitotoxic neurodegeneration. Vascular dementia, on the other hand, is affected by cerebral hypoperfusion and may profit from calcium channel blockade due to relaxation of the cerebral vasculature (Sen et al., 1993; Nimmrich and Eckert, 2013).

A completely different view of the possibility of reducing the risk of dementia due to CCBs is presented by Paris et al. (2011). The authors state that there are contradictory results on the subject in the literature, and therefore their aim was, in an experimental form (both *in vitro* and *in vivo*), to evaluate the effect of CCB directly on the production of amyloid-beta, the presence of which is associated with AD. *In vitro*, nilvadipine, nitrendipine and amlodipine were shown to be effective. *In vivo*, nilvadipine and nitrendipine were effective in amyloid-beta reduction in mice of AD models, whereas amlodipine was not. The authors propose the hypothesis that the preventive effect on the development of dementia may not be related to the antihypertensive effect itself, but to the ability of the particular substance to reduce amyloid-beta production. Nilvadipine reached the best results in mouse models, however, clinical trials have not yet been conducted on this subject. Therefore, on the basis of evidence based medicine (EBM), the authors of this article consider nitrendipine the most promising and explored dihydropyridine calcium blocking antihypertensive agent in the prevention of dementia.

*In vitro* and *in vivo* studies have been conducted both in animal models and in humans in relation to the prevention of development of dementia and the use of CCB, but the results are not uniform. In general, ineffectiveness or even worsening of memory have been observed with nifedipine, diltiazem, verapamil and amlodipine (Skinner et al., 1992; Maxwell et al., 1999; Bachmeier et al., 2011; Paris et al., 2011). Such conclusions do not concern one of the CCBs, specifically, nitrendipine. There are no references to ineffectiveness or negative effects on cognitive function, on the contrary, some studies describe it the most promising agent (Staessen et al., 1997; Forette et al., 1998, 2002; Bachmeier et al., 2011; Paris et al., 2011). For the sake of completeness, it is important to emphasize that no current guidelines for the treatment or prevention of dementia recommend that the CCB should be used.

## Nitrendipine and Dementias

Valid and respected evidence of nitrendipine efficacy was published in the SYST-EUR study (Staessen et al., 1997). This is one of the most important cardiovascular studies of the 90s aimed at verifying the efficacy of nitrendipine in the treatment of isolated systolic hypertension in old age. The number of subjects was 4,695. The conclusion of the study was, apart from a significant drop in blood pressure, that there was a significant decrease in cardiovascular complications in patients over 60 years of age with isolated systolic hypertension treated with nitrendipine. In 1,000 patients treated for 5 years, 29 cases of stroke and 53 cases of significant cardiovascular complications were prevented. A separate part of the study, called SYST-EUR-DEMENTIA, was to investigate whether antihypertensive therapy with nitrendipine may function as a prevention of vascular dementia in older patients with the above-mentioned indication (Forette et al., 1998). The total number of patients included in this observation was 2,418 (1,180 placebo, 1,238 treated). These were patients from 106 centers in 19 European countries. Initially, patients were screened by an MMSE questionnaire (at least 24 out of 30). In the course of the study, the questionnaire was repeatedly investigated and, in the case of a score of 23 and less, the presence of dementia was stated, which was further verified and its type (DSM-III-R, CT, Hachinski) was specified. In the study itself, 32 cases of dementia (23 Alzheimer’s, 2 vascular, 7 mixed) were identified, of which 21 were in the placebo group and only 11 in the group treated with nitrendipine. The authors conclude that the treatment with nitrendipine reduces the incidence of dementia by 50%, i.e., a reduction in incidence from 7.7 to 3.8 cases per 1,000 patients/year.

The same authors decided to further extend this study (Forette et al., 2002) in order to refine the results obtained in the previous study and to compare them with the results of the patients who were newly included in the study as SYST-EUR 2. The examination methods were the same as in the previous case. In total, 2,902 patients (1,417 placebo, 1,485 treated). The authors confirmed the previously achieved results and that nitrendipine was able to reduce the incidence of dementia (Alzheimer’s, vascular and mixed) by 55%, i.e., treatment of 1,000 patients 5 years of nitrendipine may prevent 20 cases of dementia.

## Discussion and Conclusion

Dementia has become a significant health and social phenomenon worldwide, affecting the whole society by means of the patients’ families as the dominant disability of the senior population. Nevertheless, it still appears that neither in the health care policy nor in the pre-graduate and postgraduate education of healthcare workers, this serious issue gets the attention it deserves. The biggest problem of diagnostics appears to be the lack of care and delayed identification of patients with dementia in primary care. According to the recent meta-analysis, GPs are able to recognize 74% of developed dementia and only 40% of mild cognitive disorders or dementia (Mitchell et al., 2011). Given the present limited treatment options, early detection and early onset of treatment are the key factors in delaying disability and institutionalizing dementia.

As far as the clinical studies exploring the connection between hypertension and dementia are concerned, at present, there are several randomized, placebo-controlled trials available on this topic. In the SHEP study (SHEP Cooperative Research Group, 1991) with chlortalidone (multicenter, randomized, double-blind, placebo-controlled, 4,736 patients over 60 years of age, mean age 70 years, 57% of females), treatment led to a marked decline in cerebral vascular events. However, there was no effect on cognitive impairment and dementia in patients treated actively despite the reduction of blood pressure and cerebral vascular events. In the HYVET-COG study (Peters et al., 2008; multicenter, randomized, double blind, placebo-controlled trial, 3,336 patients over 80 years of age, mean age 83.5 years, 61% of women) with indapamide, there was a trend towards the decreased incidence of dementia, but this decline was not statistically significant. The MRC study (Lever and Brennan, 1993; Prince et al., 1994; multicenter, randomized, placebo-controlled, 4,396 patients aged 65–74 years, combination therapy with diuretics and β-blockers) did not significantly reduce the incidence of dementia and the incidence of impaired cognitive function in a subset of patients despite the fact that the treatment also led to a reduction in blood pressure over the placebo group by 10.6/5.5 mm Hg. In the SCOPE study (Lithell et al., 2003; multicenter, double-blind, randomized, placebo-controlled, 4,964 patients aged 70–89), candesartan (+ hydrochlorothiazide) and its possible preventive effect on cognitive function were studied. Conclusion is that cognitive function despite the blood pressure decrease was not affected by candesartan compared to placebo. Positive results can be found in the PROGRESS study (Neal and MacMahon, 1995; multicenter, double-blind, randomized, placebo-controlled, 6,000 patients) in which perindopril (in combination with indapamide) was used in patients with a history of stroke or transient ischemic disorder. The results showed that the risk of dementia had been significantly reduced by 34%. The results of the SYST-EUR trial (Forette et al., 1998, 2002), in which nitrendipine reduced the incidence of dementia by more than 50%, showed the most significant antihypertensive effect in the prevention of dementia. However, for the sake of completeness, it should be added that part of the patients in the SYST-EUR study were treated with combined treatment with nitrendipine with enalapril or indapamide, and this fact could lead to a partial bias in the results.

Thus, nitrendipine is an effective and safe antihypertensive agent in both monotherapy and combination therapy, the efficacy of which has been shown to reduce the incidence of cardiovascular events on the basis of EBM, and therefore nitrendipine treatment is advantageous in elderly patients with systolic hypertension. These patients are particularly at risk of developing cardiovascular events. In addition, the SYST-EUR study demonstrated its preventive ability to reduce the risk of dementia. The results of the detected research studies indicate that nitrendipine is able to reduce the incidence of dementia (AD, vascular and mixed) by 55%. Therefore, it may be thought that nitrendipine alone or in combination would be appropriate in hypertonic patients of productive age (ideally with onset systolic hypertension) and at increased risk for dementia, for example, with a family history (Staessen et al., 1997; Forette et al., 1998, 2002).

What are the possible explanations for these extremely positive and preventive effects of nitrendipine in dementia therapy? Nitrendipine penetrates the blood-brain barrier and blocks the uncontrolled influx of calcium into the neurons. Nitrendipine is primarily bound to brain sites affected by Alzheimer’s dementia, and it is known that β-amyloid, which is accumulated in the brain tissue of elderly people with Alzheimer’s dementia and may be associated with impairment of cognitive function, increases intraneuronal calcium concentration and may sensitize brain to neurotoxin (Hockerman et al., 1997). Another, no less important role, is certainly played by a systemic reduction in blood pressure. Thus, the action of nitrendipine may be very beneficial in this overall context (Bachmeier et al., 2011; Paris et al., 2011). Surprisingly, at the moment there are no new studies with nitrendipine, which would further research the positive impact of this therapy on AD.

However, what has not yet been explained is the temporal link between hypertension and dementia due to the long-time intervals between hypertension and the development of dementia (see Parsons et al., 2016). Therefore, further research in this area is needed. Nevertheless, the treatment of hypertension with CCB is undoubtedly justified due to the proven positive effects on cardiovascular morbidity and mortality, despite its potential positive effect on cognitive function (Staessen et al., 2011).

The research indicates that currently there are 112 agents in the AD treatment pipeline. In addition, there are 26 agents in 35 trials in phase III, 63 agents in 75 trials in phase II, and 23 agents in 25 trials in phase I. A review of the mechanisms of actions of the agents in the pipeline demonstrates that 63% are disease-modifying therapies, 22% are symptomatic cognitive enhancers, and 12% are symptomatic agents focusing on neuropsychiatric and behavioral changes. Of the antihypertensive drugs, only telmisarin (phase I) and candesartan (phase II) are in clinical trials (Cummings et al., 2018). At present, there are no novel studies on the effect of the nitrendipine therapy on patients with AD. Therefore, future research should focus on this issue.

Furthermore, due to the rise of new dementia cases and consequently, economic and social burden on patients, their caregivers and the whole healthcare system, there is an urgent need to find new treatment for dementia. The treatment of hypertension with CCB showed that there might be a way to repurpose drugs for new dementia therapies (Appleby et al., 2013).

## Author Contributions

MN, BK and MV equally contributed to the drafting, analyses and final version of the whole manuscript. All authors read and approved the final manuscript.

## Conflict of Interest Statement

The authors declare that the research was conducted in the absence of any commercial or financial relationships that could be construed as a potential conflict of interest.
